# Dr. M. M. Meyers

**Published:** 1887-03

**Authors:** 


PERSONAL.—Dr. M. M. Myers, late of Lyons, has removed to Waco, to practice. The people of that wide-awake city have cause to be proud of the array of medical talent already centered there, and will find Dr. Myers a valuable acquisition. We bespeak for him a cordial welcome and wish him much success.

